# Proteasome subunit expression analysis and chemosensitivity in relapsed paediatric acute leukaemia patients receiving bortezomib-containing chemotherapy

**DOI:** 10.1186/s13045-016-0312-z

**Published:** 2016-09-06

**Authors:** Denise Niewerth, Gertjan J. L. Kaspers, Gerrit Jansen, Johan van Meerloo, Sonja Zweegman, Gaye Jenkins, James A. Whitlock, Stephen P. Hunger, Xiaomin Lu, Todd A. Alonzo, Peter M. van de Ven, Terzah M. Horton, Jacqueline Cloos

**Affiliations:** 1 Department of Pediatric Oncology/Hematology, VU University Medical Center, Amsterdam, The Netherlands; 2 Department of Amsterdam Rheumatology & Immunology Center, VU University Medical Center, Amsterdam, The Netherlands; 3 Department of Hematology, VU University Medical Center, De Boelelaan 1117, 1081 HV Amsterdam, The Netherlands; 4 Department of Pediatrics, Texas Children’s Cancer and Hematology Centers, Baylor College of Medicine, Houston, TX USA; 5 Department of Pediatrics, Hospital for Sick Children, University of Toronto, Toronto, Canada; 6 Department of Pediatrics, University of Colorado Health Sciences Center, Denver, CO USA; 7 COG Operations Office, Arcadia, CA USA; 8 Department of Preventive Medicine, University of Southern California, Los Angeles, CA USA; 9 Department of Epidemiology and Biostatistics, VU University Medical Center, Amsterdam, The Netherlands

**Keywords:** Pediatric acute leukaemia, Bortezomib, Proteasome inhibitor, Immunoproteasome

## Abstract

**Background:**

Drug combinations of the proteasome inhibitor bortezomib with cytotoxic chemotherapy are currently evaluated in phase 2 and 3 trials for the treatment of paediatric acute myeloid leukaemia (AML) and acute lymphocytic leukaemia (ALL).

**Methods:**

We investigated whether expression ratios of immunoproteasome to constitutive proteasome in leukaemic cells correlated with response to bortezomib-containing re-induction chemotherapy in patients with relapsed and refractory acute leukaemia, enrolled in two Children’s Oncology Group phase 2 trials of bortezomib for ALL (COG-AALL07P1) and AML (COG-AAML07P1). Expression of proteasome subunits was examined in 72 patient samples (ALL *n* = 60, AML *n* = 12) obtained before start of therapy. Statistical significance between groups was determined by Mann-Whitney *U* test.

**Results:**

Ratios of immunoproteasome to constitutive proteasome subunit expression were significantly higher in pre-B ALL cells than in AML cells for both β5i/β5 and β1i/β1 subunits (*p* = 0.004 and *p* < 0.001). These ratios correlated with therapy response in AML patients; β1i/β1 ratios were significantly higher (*p* = 0.028) between patients who did (*n* = 4) and did not reach complete remission (CR) (*n* = 8), although for β5i/β5 ratios, this did not reach significance. For ALL patients, the subunit ratios were also higher for patients who showed a good early response to therapy but this relation was not statistically significant. Overall, for this study, the patients were treated with combination therapy, so response was not only attributed to proteasome inhibition. Moreover, the leukaemic blast cells were not purified for these samples.

**Conclusions:**

These first ex vivo results encourage further studies into relative proteasome subunit expression to improve proteasome inhibition-containing therapy and as a potential indicator of bortezomib response in acute leukaemia.

**Electronic supplementary material:**

The online version of this article (doi:10.1186/s13045-016-0312-z) contains supplementary material, which is available to authorized users.

## Background

The survival of paediatric patients with leukaemia has greatly improved in recent decades. However, 10–30 % of patients still relapse, and outcome after relapse remains poor, with remission rates as low as 10–40 % for poor prognostic subtypes [1, 2]. Hence, there is a need for improvement in therapeutic options for these patients. Based on the proven clinical activity in multiple myeloma (MM) [3] and mantle cell lymphoma [4], as well as encouraging preclinical data from our lab [5] and the Paediatric Preclinical Testing Program [6], the proteasome inhibitor (PI) bortezomib is currently undergoing clinical evaluation in paediatric leukaemia [7, 8].

Proteasomes are large, multi-subunit complexes located in both the cytosol and nucleus and are responsible for the degradation of the majority of intracellular proteins. In addition to constitutive proteasomes (β1 + β2 + β5), the immunoproteasome (β1i + β2i + β5i) represents a proteasome variant that is predominantly expressed in haematopoietic cells [9–11]. The immunoproteasomes produce peptides similar to constitutive proteasomes. Although the exact role of the immunoproteasome has yet to be defined, the peptide fragments produced by the immunoproteasome are thought to play a role in antigen presentation to MHC class I antigens (reviewed in [12]). Disruption of proteasome activity results in rapid accumulation of toxic regulatory proteins in the cell that results in endoplasmic reticulum (ER) stress and activates the unfolded protein response (UPR), which blocks protein translation and initiates alternative degradation pathways in stress situations [13]. Another mechanism of action of bortezomib is thought to inhibit NF-kB chemotherapy-induced pro-survival pathways in leukaemic blasts by blocking the proteasomal degradation of NF-kB’s natural inhibitor, IkB [14].

Although bortezomib displays only modest single-agent activity in children [15] and adults [16–18], it has additive or synergistic interactions with cytotoxic agents commonly used to treat acute leukaemia including glucocorticoids, vincristine [5] and anthracyclines [19]. Phase 1/2 studies combining bortezomib with standard induction/consolidation regimens have shown promising clinical activity in both adult AML [20–22] and paediatric ALL [8, 23]. However, bortezomib did not improve complete remission (CR) rate or overall survival in a paediatric AML cohort [7]. Thus, the clinical impact of PI therapy in combination with cytotoxic chemotherapy has not yet been fully assessed.

Despite the encouraging results of bortezomib in early-phase clinical trials, primary or acquired resistance to bortezomib may limit its efficacy [24, 25] and thus identifying those patients who respond to bortezomib-containing therapy is of great clinical relevance. Recently, we reported that higher ratios of immunoproteasome to constitutive proteasome protein expression in paediatric ALL and AML leukaemia cells at diagnosis predicted ex vivo sensitivity to bortezomib and other PIs [10]. The importance of subunit assembly for PI sensitivity was further investigated in leukaemia cell lines, revealing that interferon-γ-induced upregulation of immunoproteasome subunit expression and concomitant downregulation of constitutive subunit expression, markedly sensitizing bortezomib-resistant cells to PIs, with the β5i being the most important subunit involved in this process [26].

Here, we explored whether ratios of immunoproteasome to constitutive proteasome protein expression correlate with bortezomib response in paediatric leukaemia patients treated with bortezomib-containing re-induction chemotherapy. Specifically, paediatric patients with acute leukaemia in first relapse were enrolled in two Children’s Oncology Group (COG) phase 2 trials of bortezomib combined with re-induction chemotherapy for paediatric ALL (AALL07P1) or paediatric AML (AAML07P1). In addition to (immuno) proteasome profiling, we also examined leukaemia cells for bortezomib-induced alterations in NF-kB activity.

## Methods

### Patients

Pre-treatment, snap-frozen peripheral blood mononuclear cells (PBMCs) or mononuclear cells isolated from the bone marrow were obtained from 84 patients enrolled in either the ALL (COG-AALL07P1; NCT00873093) or AML (COG-AAML07P1; NCT00666588) clinical trials between 2009 and 2013. Since no differences in overall survival between the two randomization arms in AAML07P1 were observed [7], no distinction was made between AML patients receiving etoposide or idarubicin. Informed consent was obtained from the patient or their parent(s), and the studies have been performed according to the Declaration of Helsinki. Assent, as appropriate, were obtained in accordance with the US National Cancer Institute, and the study was approved by the relevant COG committees, CTEP and the paediatric central IRB in accordance with institutional policies for human subjects’ research. Mononuclear cells were isolated using Ficoll density centrifugation prior to proteasome and NF-kB analysis. Only samples with blast percentages >20 % were included in the analyses. Seventy-two patient samples were available for proteasome subunit expression analysis (60 ALL and 12 AML), and 32 patient samples (22 ALL and 10 AML) for proteasome subunit catalytic activity. Forty-eight patient samples (36 ALL and 12 AML) were evaluable for NF-kB analysis. Table 1 summarizes patient demographics.

**Table 1:** Patient demographics

	ALL (*n* = 68)	AML (*n* = 16)
Age, years median (range)	9.9 (1.5–23.7)	9.6 (1.0–19.6)
Sex		
Female	22	9
Male	46	7
Race		
Caucasian	47	10
Black-American	9	1
Asian/Indian	5	4
Native American/Hawaiian	1	0
Other/unknown	6	1
Ethnicity		
Hispanic	21	3
Non-Hispanic	46	11
Unknown	1	2
Performance score (ECOG)		
0	56	9
1	9	7
2	3	0
WBC median, 10^9^/L (range)	12.8 (0.3–201.1)	11.6 (0.5–61.8)
ALL histologic subtype		
pre-B ALL	52	
T-ALL	16	
AML type		
Relapsed		11
Secondary		5
AML: prior BMT		
No		12
Yes		4

### Protein expression/Western blotting

Antibodies to proteasome subunits β1, β2, β5, β1i and β5i were purchased from Enzo Life Sciences (Farmingdale, NY, USA), and the IRDye infrared-labelled secondary antibodies were from LI-COR Biosciences (Lincoln, NE, USA). In addition, anti-actin (clone C4) was purchased from Millipore (Temecula, CA, USA). Protein expression levels of constitutive proteasome subunits β5 (PW-8895) and β1 (PW-8140) and immunoproteasome subunits β5i (PW-8845) and β1i (PW-8345) were determined by Western blot analysis as previously described [10, 26, 27]. Protein bands were quantified by Odyssey software, corrected for background, and normalized with β-actin. Subunit expression between patient samples on different gels was normalized using subunit expression in the leukaemic T-ALL cell line CCRF-CEM [27].

### Subunit-specific β5 and β5i proteasome catalytic activities

Subunit-specific proteasome catalytic activity of β5 (Ac-WLA-AMC) and β5i (Ac-ANW-AMC) was measured using fluorogenic substrates [28] as previously described [26] in cell extracts from 22 ALL and 10 AML samples.

### Active p65 NF-kB by ELISA

PBMCs (*n* = 48) were isolated from whole blood of patients prior to bortezomib treatment and at 6 and 24 h after the first bortezomib dose. Nuclear lysates were prepared, and active p65 NF-kB was determined by ELISA (Active Motif, Carlsbad, CA) as previously described [15]. NF-kB ELISA activity was performed in triplicate and averages used for comparisons over time and blast percentage ranged from 21 to 93 % (median 53 %) for ALL (*n* = 36) and from 22 to 85 % (median 52 %) for AML (*n* = 12).

### Statistics

Correlations were calculated by determining Spearman’s rank correlation coefficients. Statistical significance between groups was determined by Mann-Whitney *U* test. Logistic regression analysis was used to determine whether the measured predictor variables were associated with response to therapy, both with and without correction for leukaemia type (ALL/AML), blast percentages, gender, age and white blood cell count. All statistical analyses were performed using IBM-SPSS (version 20.0). A two-sided significance level of 0.05 was used in all statistical analyses.

## Results

### Proteasome subunit expression in relapsed childhood acute leukaemia samples

**Fig. 1:**
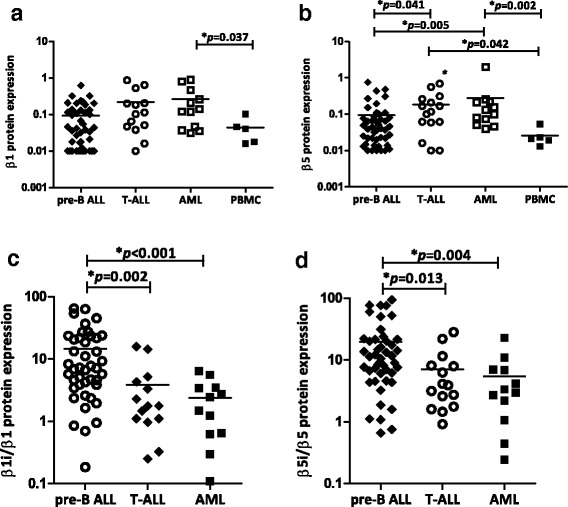
Proteasome subunit protein expression in relapsed childhood ALL and AML. Comparison of **a** constitutive subunit β1 **b** and constitutive subunit β5 expression for pre-B ALL (*n* = 46), T-ALL (*n* = 14) and AML (*n* = 12) patient samples and PBMCs from healthy adult volunteers (*n* = 5). **c** The ratio of paired subunits β1i/β1. **d** The ratio of paired subunits β5i/β5, within each patient sample for pre-B ALL (*n* = 46), T-ALL (*n* = 14) and AML (*n* = 12) patient samples. Protein expression was assessed by Western blotting and expressed as relative quantifications of subunit expression (ratio proteasome subunit/β-actin based on loading of 15 μg total protein, normalized to CEM). The *line* denotes the mean. Significant *p* values are noted (Mann-Whitney *U* test)

Consistent with our previous preliminary findings [10], in this cohort of 60 relapsed ALL (46 pre-B ALL, 14 T-ALL) and 12 relapsed AML patients, AML blasts did not differ from pre-B ALL or T-ALL blasts in constitutive β1 subunit expression (Fig. 1a) while both AML blasts and T-ALL blasts had higher β5 subunit expression than pre-B ALL blasts (*p* = 0.005 and *p* = 0.041, respectively; Fig. 1b; Table 2). In this subset of samples, ALL samples had a significantly higher blast percentage (median 84 %) than AML samples (median 59 %) (*p* < 0.001). In order to determine if the normal cells in the AML samples contribute to the proteasome subunit composition, proteasome subunit expression of PBMCs from healthy adult volunteers (*n* = 5) were compared to pre-B ALL and AML samples and revealed that the median constitutive β1 and β5 expression are both significantly lower in PBMCs compared to AML patient samples (β1 *p* = 0.037 and β5 *p* = 0.002), indicating that the normal cells in leukaemia samples do not significantly contribute to the proteasome subunit expression comparisons (Fig. 1a, b). In contrast, pre-B ALL blasts had higher immunoproteasome subunit β1i expression than AML blasts (*p* = 0.048), while no significant difference was observed between ALL and AML for the expression of immunoproteasome subunit β5i (Additional file 1: Figure S1A, B). As a consequence, median ratios of both β1i/β1 and β5i/β5 expression were significantly higher in pre-B ALL patients than in T-ALL patients (*p* = 0.002 for β1i/β1 and *p* = 0.013 for β5i/β5) and in AML patients (*p* < 0.001 for β1i/β1 and *p* = 0.004 for β5i/β5 Fig. 1c, d; Table 2). Median blast percentages did not differ between T-ALL (median 81 %) and pre-B ALL (median 86 %).

**Table 2:** Median proteasome subunit protein expression and NF-kB activity in paediatric acute leukaemia patients

	β5 (range)	β1 (range)	β5i (range)	β1i (range)	β5i/β5 (range)	β1i/β1 (range)	NF-kB activity
ALL	0.055 (0.01–0.76)	0.05 (0.01–0.86)	0.46 (0.06–3.43)	0.24 (0.07–1.45)	8.2 (0.66–94.5)	5.2 (0.18–64.7)	128.1 (11.8–1025.7)
CR	0.045 (0.01–0.76)	0.05 (0.01–0.64)	0.47 (0.06–2.08)	0.31 (0.07–1.06)	8.55 (0.76–94.5)	6.8 (0.19–64.7)	253.5 (11.8–1022.2)
No CR	0.065 (0.01–0.56)	0.1 (0.01–0.86)	0.38 (0.13–3.43)	0.22 (0.07–1.45)	6.7 (0.66–52.5)	3.4 (0.25–27.2)	73.0 (24.5–1025.7)
Pre-B ALL	0.04 (0.01–0.76)	0.04 (0.01–0.62)	0.46 (0.06–3.43)	0.33 (0.07–1.45)	11.4 (0.66–94.5)	7.1 (0.19–64.7)	90.2 (11.8–1025.7)
CR	0.03 (0.01–0.76)	0.04 (0.01–0.62)	0.57 (0.06–2.08)	0.43 (0.07–1.06)	13.4 (0.76–94.5)	7.8 (0.19–64.7)	89.9 (11.8–1022.2)
No CR	0.06 (0.01–0.49)	0.1 (0.01–0.32)	0.42 (0.13–3.43)	0.23 (0.12–1.45)	9.0 (0.66–52.5)	4.2 (0.7–27.2)	98.9 (24.5–1025.7)
T-ALL	0.12 (0.01–0.69)	0.11 (0.01–0.86)	0.44 (0.17–1.12)	0.21 (0.07–0.58)	3.9 (0.92–28.5)	1.77 (0.25–15.9)	333.7 (35.3–625.3)
CR	0.21 (0.06–0.56)	0.1 (0.04–0.86)	0.37 (0.17–0.82)	0.11 (0.07–0.21)	1.78 (1.46–2.74)	1.1 (0.25–1.77)	393.5 (262.5–625.3)
No CR	0.1 (0.01–0.69)	0.11 (0.01–0.64)	0.46 (0.18–1.12)	0.22 (0.16–0.58)	5.2 (0.92–28.5)	2.28 (0.33–15.9)	37.9 (35.3–40.5)
AML	0.12 (0.04–1.98)	0.13 (0.03–0.88)	0.41 (0.09–1.08)	0.17 (0.1–0.64)	3.2 (0.24–23.0)	1.97 (0.11–6.4)	524.5 (35.3–1323.7)
CR	0.089 (0.04–0.21)	0.042 (0.031–0.26)	0.23 (0.09–0.92)	0.23 (0.13–0.64)	4.0 (0.45–23.0)	4.5 (2.4–6.3)^a^	804.1 (284.5–1323.7)
No CR	0.13 (0.05–1.98)	0.18 (0.04–0.88)	0.52 (0.22–1.1)	0.16 (0.1–0.49)	3.2 (0.24–11.1)	0.94 (0.11–3.5)	524.5 (35.3–1246.5)

Please note that Western blot data depict relative quantifications of subunit expression (ratio proteasome subunit/β-actin based on loading of 15 μg total protein, normalized to CEM). Quantifications were based on one Western blot analysis per sample. For three patients multiple replicate quantifications of subunits could be performed with a mean SD of <26 %. Lower limit of detection of protein bands quantification by Odyssey software; set to 0.01 AU for β5 and β1, 0.06 AU for β5i and 0.07 AU for β1i

Pre-B ALL *n* = 45 patient samples for β5 and β5i, *n* = 43 for β1 and β1i, T-ALL *n* = 15 for β5 and β5i, *n* = 14 for β1 and β1i, AML *n* = 12 patient samples were used

*CR* complete remission

### Proteasome subunit expression correlations with response to re-induction therapy

**Fig. 2:**
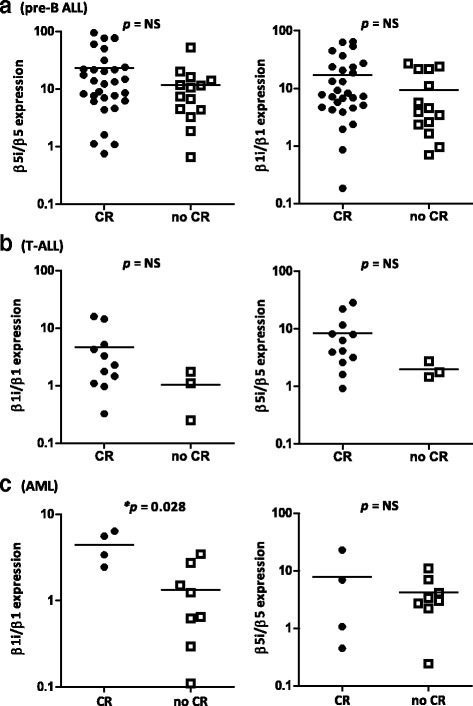
Ratio proteasome subunit protein expression in patients who achieved complete remission versus patients who did not, dissected by leukaemia subtype. Ratios of immunoproteasome/constitutive proteasome protein expression in **a** AML patients, **b** pre-B ALL patients and **c** T-ALL patients that achieved CR versus patients who did not achieve CR, determined by Western blot analysis after bortezomib-containing re-induction therapy. The *line* denotes the mean

Next, we compared differences in proteasome protein subunit expression between patients that attained CR vs. no CR after the first re-induction therapy block. When samples were subdivided by leukaemia subtype, a statistically significant difference was detected in median β1i/β1 ratios between CR (*n* = 4) and no CR (*n* = 8) in patients with AML (*p* = 0.028, Fig. 2a; Table 2). No significant differences were detected for median β5i/β5 ratios, also not within ALL subgroups (Fig. 2b, c). Median blast percentages did not differ between patients that attained CR and patients that did not attain CR. Despite the fact that pre-treatment ratios of β5i/β5 or β1i/β1 were related to achievement of CR, these parameters were not strong enough to independently predict treatment response in either univariable logistic regression analyses or after controlling for blast percentage, leukaemia subtype, gender, age and white blood cell count in multi-variable analysis (data not shown).

### Proteasome catalytic activity

**Fig. 3:**
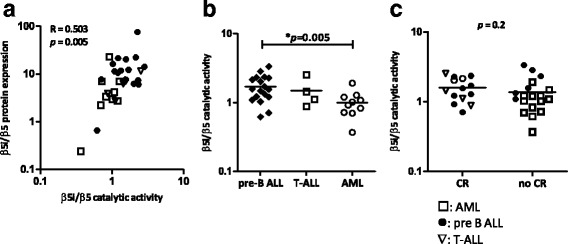
Proteasome subunit catalytic activity in relapsed childhood ALL and AML patients. **a** Correlation of β5i/β5 protein expression and β5i/β5 catalytic activity ratios. **b** β5i/β5 catalytic activity ratios in pre-B ALL patients (*n* = 18) compared to T-ALL patients (*n* = 4) and AML patients (*n* = 10), and **c** β5i/β5 catalytic activity ratios in patients who achieved complete remission (*n* = 14) versus those who did not (*n* = 18). *Closed circles* represent pre-B ALL patients, *open triangles* T-ALL patients and *open squares* AML patients. The *line* denotes the mean

There was a good correlation between proteasome subunit expression and catalytic activity for β5i/β5 (*R* = 0.503 *p* = 0.005, Fig. 3a). The median activity ratio of β5i/β5 differed significantly between pre-B ALL (*n* = 18) and AML (*n* = 10) patients (*p* = 0.005, Fig. 3b) while the individual subunits β5 and β5i did not differ (data not shown). In addition to proteasome subunit expression ratios, median β5i/β5 subunit activity ratios were not higher in patients who reached CR (*n* = 14) compared to patients who did not reach CR (*n* = 18) (Fig. 3c). Median blast percentages did not differ statistically between CR patients (median 81 %) and no CR patients (median 71 %).

### The effect of bortezomib treatment on NF-kB activity

**Fig. 4:**
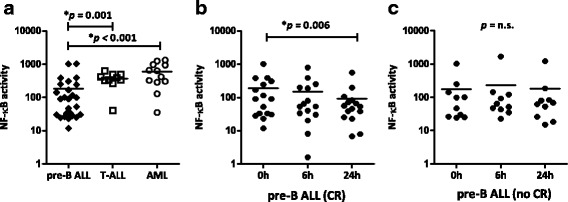
Baseline NF-kB activity between ALL and AML patients and impact of bortezomib treatment. Pre-treatment NF-kB activity measurements determined by p65-ELISA in PBMC of acute leukaemia patients comparing **a** NF-kB activity of pre-B ALL (*n* = 26), T-ALL (*n* = 10) and AML (*n* = 12) patients, and **b** NF-kB activity prior to treatment compared to 6 and 24 h after bortezomib-containing chemotherapy in pre-B ALL patients that reached CR (*n* = 16), and **c** pre-B ALL patients that did not reach CR (*n* = 10). The *line* denotes the mean

We also examined NF-kB activity in leukaemia cells in response to bortezomib-containing chemotherapy. NF-kB activity was evaluated in PBMCs of 36 ALL patients (26 pre-B ALL and 10 T-ALL) and 12 AML patients at three time points: *T* = 0, *T* = 6 and *T* = 24 h after the first bortezomib administration. Median pre-treatment NF-kB activity was significantly lower in pre-B ALL versus both T-ALL patients (*p* = 0.001) and AML patients (*p* < 0.001) (Fig. 4a, Table 2), while blast percentages were similar for these groups. Twenty-four hours after bortezomib treatment, pre-B ALL patients who attained a CR (*n* = 16) displayed a significant decrease in median NF-kB activity compared to pre-treatment levels (*p* = 0.006, Fig. 4b). In contrast, pre-B ALL patients who did not attain a complete remission (*n* = 10) after the first bortezomib cycle showed unaltered NF-kB activity after bortezomib treatment (Fig. 4c). Overall, however, changes in NF-kB activity during treatment with bortezomib-containing chemotherapy were not significantly associated with achievement of post-re-induction CR.

## Discussion

Recently, on the basis of an ex vivo study with acute leukaemia clinical specimen at diagnosis, we provided evidence that ratios of immunoproteasome subunits to constitutive subunits correlate with sensitivity of acute leukaemia cells to PIs [10]. The current research was set up to validate this in a distinct, prospectively collected sample set from patients enrolled on two COG clinical trials, which is the first to study potential biomarkers that may predict clinical sensitivity to bortezomib-containing treatment regimens.

Although approved and efficacious for treatment of MM, bortezomib treatment does not lead to response in all MM patients due to emergence of drug resistance [24]. Conceivably, resistance phenomena could also limit the clinical utility of this agent in relapsed paediatric leukaemia. Hence, parameters that could indicate the likelihood of clinical response to PI therapy would provide a mechanism to determine which patients are likely to benefit from the addition of PI therapy to their re-induction regimen. Several mechanisms have been reported to underlie bortezomib resistance [29, 30]. Bortezomib-resistant cell lines are frequently characterized by mutations in the *PSMB5* gene encoding the β5 subunit [27, 31]. To date, however, no *PSMB5* mutations have been found in patients clinically resistant to bortezomib [32–34]. Also in the current study, no *PSMB5*-associated mutations in exon 2 of the gene were identified in either end of induction (*n* = 15) or relapse (*n* = 3) samples (data not shown). This implies that, in acute leukaemia, β5 mutations make only a minor contribution to bortezomib resistance and that other common resistance mechanisms, such as overexpression of β5, play a more important role [27, 29, 31, 35]. Confirming our earlier ex vivo work in newly diagnosed patient samples [10], the current study shows that constitutive (β5 and β1) proteasome subunit expression was significantly lower in patients with relapsed ALL vs. AML, whereas β1i and β5i immunoproteasome subunit expression was higher (though it did not reach significance). These results were recently confirmed by de Bruin et al. [36], who showed by labelling analysis of primary patient cells that B cell ALL patients have higher immune/constitutive subunit expression ratios than AML cells and T-ALL cells. Given the significance of correlation between the subunit expression and catalytic activity (Fig. 3a), the subunit activity-based probes may prove useful for examining clinical specimen. Others have also reported that bortezomib sensitivity relates to proteasome expression. In particular, increased *PSMB5* mRNA expression was found in a myeloma patient who subsequently developed bortezomib resistance [33]. Moreover, bortezomib-sensitive hematologic cell lines harboured higher immunoproteasome expression levels compared to relatively bortezomib-resistant solid-tumour cell lines [37]. Lastly, a higher β2/β1 + β5 activity ratio correlated with higher bortezomib sensitivity in a cell line panel of hematologic malignancies [38]. In a recent study, we showed that interferon-γ-induced upregulation of immunoproteasome expression and concurrent constitutive proteasome subunit downregulation in bortezomib-resistant hematologic tumour cell lines resulted in the sensitization for PI treatment [26]. Consistently, knockdown of constitutive β5 in the AML cell line THP1 resulted in increased sensitivity to bortezomib [31].

NF-kB activity was evaluated by ELISA in 26 pre-B ALL, 10 T-ALL and 12 AML samples after the first dose of bortezomib. NF-kB is constitutively active in the majority of ALL patients [23, 39] and AML patients [40]. Though a significant decline in NF-kB activity was observed in pre-B ALL patients 24 h after the first bortezomib dose, this decline in NF-kB activity did not correlate with bortezomib response. Of note, Magrangeas et al. [41] found that low pre-treatment levels of NF-kB were associated with a higher response rate to bortezomib-based induction in newly diagnosed MM, but we were unable to find a similar correlation between pre-treatment NF-kB activity and bortezomib. Overall, in our current study, changes in NF-kB activity during treatment with bortezomib-containing chemotherapy were not associated with achievement of post-induction CR, and neither pre-treatment NF-kB activity nor decreases in NF-kB activity after 24 h correlated with clinical response.

This current cohort provided the first set of samples collected from patients who received bortezomib treatment and could serve as a potential validation cohort for our former test cohort [10]. Since the design of this add-on study was not yet optimized for the proteasome-based assays, there are some limitations that have to be considered. First, although only samples that contained >20 % of blasts were used, there was a large variation in blast percentage between samples (mean 72 % ± 23 %). To address the impact of normal cells, we measured proteasome subunit expression levels in normal PBMCs and found that these had a relative minor contribution on proteasome subunit expression in leukaemic cells and thus did not largely influence our analyses. Notwithstanding this fact, purification of lymphoblast/myeloblast purification is recommended in future follow-up studies. Another important issue is the fact that patients in this study were treated with combination therapy. Hence, any outcome variable is a cumulative effect of all drugs, which can confound the relation between our measurements and bortezomib sensitivity.

## Conclusions

In summary, cells of relapsed ALL patients had significantly higher β5i/β5 and β1i/β1 proteasome subunit protein expression ratios, and lower β5 and β1 constitutive subunit expression compared to cells of relapsed AML patients. Interestingly, AML patients who achieved CR after bortezomib-containing therapy had higher pre-treatment immunoproteasome/constitutive proteasome expression ratios compared to patients who did not achieve CR. Further studies using purified blast populations are being conducted to confirm that higher ratios of immuno-/constitutive proteasome in pre-treatment ALL and AML cells are associated with an initial clinical response to bortezomib-containing re-induction treatment.

## Additional file

Additional file 1: Figure S1. Individual immunoproteasome subunit protein expression in relapsed childhood acute leukaemia patients. **Figure S2.** Proteasome subunit mRNA expression in NBM cells. **Figure S3.** Proteasome subunit catalytic activity in relapsed childhood ALL and AML patients.

## Abbreviations

AML: Acute myeloid leukaemia
ALL: Acute lymphocytic leukaemia
COG: Children’s Oncology Group
CR: Complete remission
MM: Multiple myeloma
PI: Proteasome inhibitor
ER: Endoplasmic reticulum
UPR: Unfolded protein response
