# Supplementary material for: Proteasome subunit expression analysis and chemosensitivity in relapsed paediatric acute leukaemia patients receiving bortezomib-containing chemotherapy
Source: J Hematol Oncol. 2016 Sep 6;9(1):82. doi: 10.1186/s13045-016-0312-z (PMC5011854; doi:10.1186/s13045-016-0312-z)
Supplement: Additional file 1: Figure S1. — Individual immunoproteasome subunit protein expression in relapsed childhood acute leukaemia patients. Figure S2. Proteasome subunit mRNA expression in NBM cells. Figure S3. Proteasome subunit catalytic activity in relapsed childhood ALL and AML patients. [file 13045_2016_312_MOESM1_ESM.pdf]

Supporting Information

Figure S1

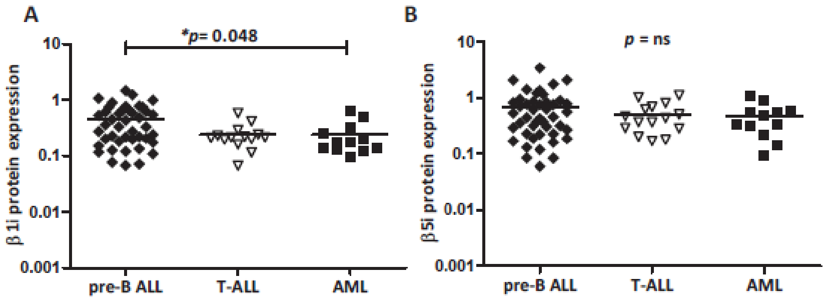

Figure S1. Individual immunoproteasome subunit protein expression in relapsed childhood acute leukaemia patients. Comparison of (**A**) immunoproteasome subunit β1i, and (**B**) immunoproteasome subunit β5i, between pre-B ALL, T-ALL and AML patients. Protein expression was assessed by Western blotting and expressed as relative quantifications of subunit expression (ratio proteasome subunit/β-actin based on loading of 15 µg total protein, normalized to CEM). The closed symbols represent ALL patients, the open symbols AML patients. The line denotes the mean.

Figure S2.

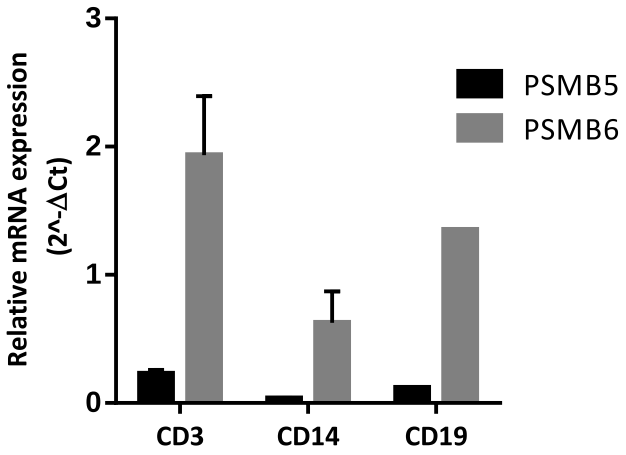

Figure S2. Proteasome subunit mRNA expression in NBM cells. Expression of *PSMB5* and *PSMB6* in sorted CD3 (T-cells), CD14 (monocytes), and CD19 (B-cells) from 2 healthy individuals determined by qRT-PCR normalized on expression of GUS housekeeping gene.

Figure S3 eventueel? AANPASSEN MET NIEUWE SAMPLES

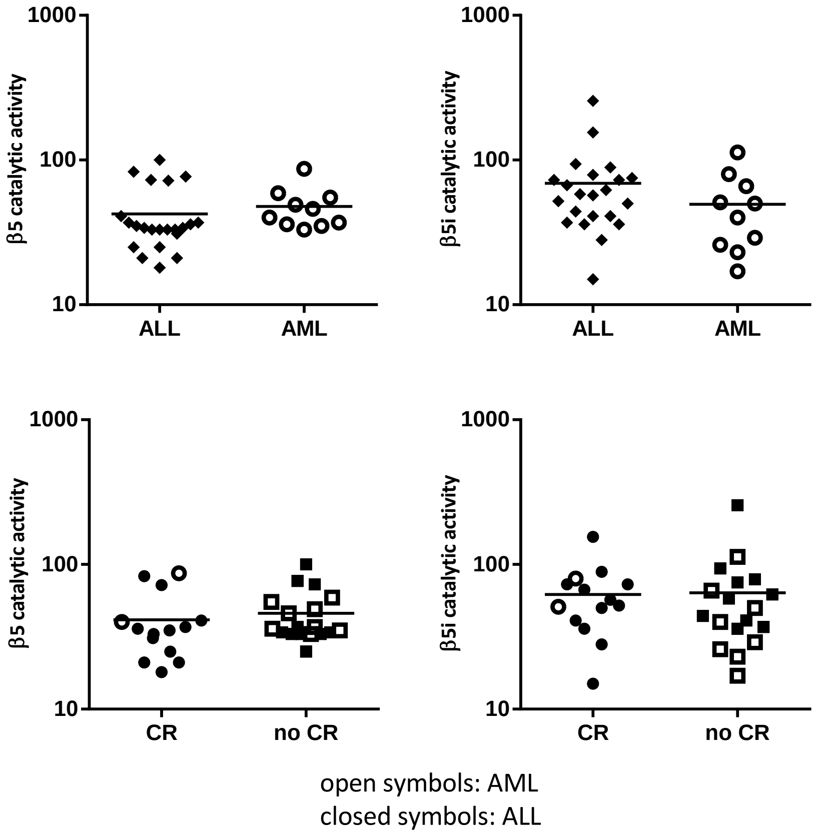

Figure S3. Proteasome subunit catalytic activity in relapsed childhood ALL and AML patients. Comparison of catalytic activity of constitutive subunit β5 and immunoproteasome subunit β5i between ALL and AML patients. The line denotes the mean for ALL (n=22) and AML (n=10).
